# Modelling heat conduction in polycrystalline hexagonal boron-nitride films

**DOI:** 10.1038/srep13228

**Published:** 2015-08-19

**Authors:** Bohayra Mortazavi, Luiz Felipe C. Pereira, Jin-Wu Jiang, Timon Rabczuk

**Affiliations:** 1 Advanced Materials Multiscale Modeling, Institute of Structural Mechanics, Bauhaus-Universität Weimar, Marienstr. 15, D-99423 Weimar, Germany; 2 Departamento de Física Teórica e Experimental, Universidade Federal do Rio Grande do Norte, Natal, 59078-970, Brazil; 3 Shanghai Institute of Applied Mathematics and Mechanics, Shanghai Key Laboratory of Mechanics in Energy Engineering, Shanghai University, 200072, People’s Republic of China; 4 Chair of Computational Mechanics, Institute of Structural Mechanics, Bauhaus University Weimar; 5 Korea University, School of Civil, Environmental and Architectural Engineering, Seoul, South Korea

## Abstract

We conducted extensive molecular dynamics simulations to investigate the thermal conductivity of polycrystalline hexagonal boron-nitride (h-BN) films. To this aim, we constructed large atomistic models of polycrystalline h-BN sheets with random and uniform grain configuration. By performing equilibrium molecular dynamics (EMD) simulations, we investigated the influence of the average grain size on the thermal conductivity of polycrystalline h-BN films at various temperatures. Using the EMD results, we constructed finite element models of polycrystalline h-BN sheets to probe the thermal conductivity of samples with larger grain sizes. Our multiscale investigations not only provide a general viewpoint regarding the heat conduction in h-BN films but also propose that polycrystalline h-BN sheets present high thermal conductivity comparable to monocrystalline sheets.

The great success of graphene^1^,^2^,^3^ with its extraordinary combination of high thermal conductivity^4^,^5^, mechanical strength^6^ and electrical properties^7^ raised an ongoing attention towards other two-dimensional (2D) materials. Numerous experimental and theoretical studies have confirmed the outstanding and broad prospects for the application of 2D materials: from flexible nanoelectronics to aerospace structures. However, the high electrical conductivity of graphene forbids its application in devices where the building blocks are required to be electrically insulating. Nevertheless, several other 2D materials with inherent semiconducting properties have been fabricated such as hexagonal boron-nitride (h-BN)^8^, molybdenum disulfide (MoS_2_)^9^, and triazine-based graphitic carbon nitride (g-C_3_N_4_) nanosheets^10^. Among the available 2D semiconducting materials h-BN possesses the highest thermal conductivity and mechanical strength^11^,^12^,^13^,^14^. Similarly to graphene, h-BN presents a honeycomb lattice in which carbon atoms are replaced with alternating pairs of boron and nitrogen atoms. Because of this similarity in atomic structure, graphene and h-BN have been proposed as candidates for the construction of hetero-structures with tuneable physical properties^15^,^16^.

Analogous to graphene, chemical vapor deposition (CVD) is the major route to produce large-scale h-BN films^17^,^18^. However, crystal growth during the CVD method yields polycrystalline structures. Therefore, CVD grown h-BN films consist of different grains which are connected by grain boundaries throughout the sample. Grains with different growth orientations match together through grain boundaries that include various types of topological defects. Like all other known materials, these defects in CVD grown h-BN films affect their intrinsic physical properties. Defects along the grain boundaries cause stress concentration and phonon scattering. In particular, these effects could lead to lower mechanical strength and thermal conductivity^19^,^20^,^21^,^22^. In polycrystalline structures, as the grain size decreases, more grain boundaries form resulting in higher defect concentrations. Accordingly, a comprehensive understanding of the effects of grain size on the physical properties of h-BN films is of crucial importance. From the experimental or the theoretical point of view, few studies exist concerning the atomic structure of grain boundaries in h-BN films. In this regard, first principle calculations suggest that grain boundaries in polycrystalline h-BN consist mainly of pentagon-heptagon pairs (5/7) and square-octagon pairs (4/8)^23^. Meanwhile, a transmission electron microscope study of CVD grown h-BN films indicates that the grain boundaries consist mainly of pentagon-heptagon pairs rather than square-octagon pairs^24^. Both theoretical calculations and experiments also reveal that defect pairs in h-BN grain boundaries include homo-nuclear boron-boron or nitrogen-nitrogen bonds, which are higher in energy than heteronuclear boron-nitrogen bonds inside the grains.

To date, heat conduction in polycrystalline h-BN films has not been addressed neither theoretically nor experimentally. Because of the complexity of such experimental studies at the nanoscale, computer simulations offer an alternative approach^25^,^26^. In the present work, we conducted classical molecular dynamics (MD) simulations to provide detailed information concerning the influence of grain size on the thermal conductivity of polycrystalline h-BN sheets. Additionally, we investigated the thermal conductivity of macroscopic films using the finite element (FE) method. In our FE models, all grain boundaries were assumed to present an effective contact conductance. This effective thermal conductance was estimated by fitting the FE results to those obtained by MD simulations. Our atomistic-continuum multiscale modelling reveals that CVD grown h-BN films can exhibit high thermal conductivity, remarkably close to that of monocrystalline, defect-free sheets.

## Results

**Figure 1:**
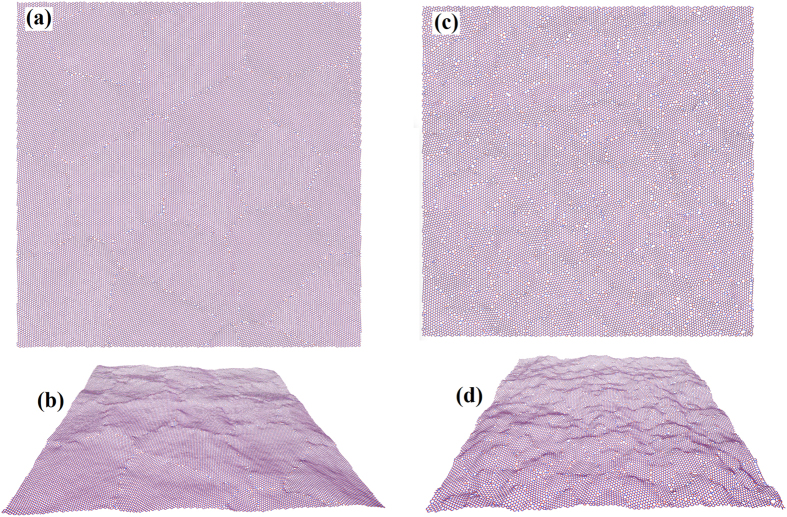
Atomistic models of periodic polycrystalline h-BN films relaxed at room temperature and zero stress using the Tersoff potential^12^. (**a**) Top and (**b**) side view of polycrystalline h-BN with an average grain size of 10 nm consisting of 62585 atoms and 16 grains. (**c**) Top and (**d**) Side view of an ultra-fine grained h-BN film with an average grain size of 2 nm including 39278 atoms and 256 individual grains. The snapshots were created with VMD^50^.

In Fig. 1 we present two snapshots of polycrystalline h-BN films obtained in our MD simulations with the Tersoff potential at 300 K. The samples correspond to equivalent grain sizes of 10 nm and 2 nm, respectively. The grain boundaries and boundary junctions are stable for temperatures in excess of 900 K, which shows that the Tersoff potential maintains the boundary structure and the defects along the boundaries thermodynamically stable even at high temperatures. For a sample with an effective grain size of 10 nm, we found that grain boundaries mainly consist of pentagon-heptagon pairs, pentagon-octagon pairs and point vacancies. In this case, the formation of square-octagon pairs is strictly limited. Nevertheless, for polycrystalline samples with an equivalent grain size of 2 nm, we could observe all combinations of square, pentagon, heptagon and octagon rings along the grain boundaries. As illustrated in the side views of the polycrystalline structures, Fig. 1(b,d), along the grain boundaries local deflection and bending of the sheets exist which is caused by topological defects. In this regard, deformations and deviations of h-BN films from planarity turn out to be more noticeable for samples with smaller grain sizes.

**Figure 2:**
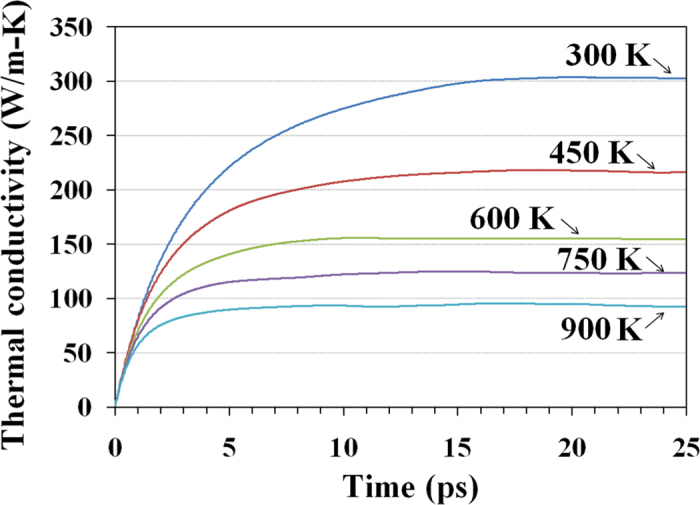
Calculated thermal conductivity of monocrystalline, defect-free h-BN as a function of correlation time for different temperatures.

In Fig. 2 we plot the calculated thermal conductivity as a function of correlation time for defect-free h-BN films at different temperatures. These results were acquired by averaging EMD results for twelve independent simulations with uncorrelated initial conditions. At room temperature, the thermal conductivity converged at a correlation time shorter than 20 ps. For monocrystalline and pristine h-BN at room temperature, our EMD results predict a thermal conductivity of 300 ± 30 W/m-K. Our simulations employ the Tersoff potential with parameters obtained by Lindsay and Broido to model heat transport in single layer and multilayer h-BN^12^. Utilising an exact numerical solution of the phonon Boltzmann transport equation, they obtained a thermal conductivity around 600 W/m-K for single layer h-BN and around 400 W/m-K for 5—layer h-BN, in good agreement with the experimental value of 390 W/m-K for in-plane thermal conductivity of bulk h-BN^11^. The discrepancy between our result and that from Ref. 12 can be partially explained by the degree of anharmonicity considered in each method. In order to solve the phonon Boltzmann equation it is necessary to ignore higher order anharmonic terms. Usually, up to third-order interatomic force constants are considered in the solution of the Boltzmann transport equation^27^,^28^,^29^,^30^, which correspond to three-phonon scattering processes. Meanwhile, in molecular dynamics simulations, at least in principle, all orders of anharmonicity are present. Therefore, it is not unusual for EMD simulations to yield thermal conductivities values lower than those obtained by solving the Boltzmann equation. We also note that in previous works the thermal conductivity of pristine single layer h-BN calculated via EMD at 300 K was predicted to be 400 W/m-K using the Tersoff potential parameterised by Sevik *et al.*^31^, and 80 W/m-K with parameters from Matsunaga *et al.*^14^,^32^. A recent experimental study on few-layer h-BN revealed that its thermal conductivity decreases when the number of layers^33^ decreases, which is in contrast with experimental observation for graphene sheets^4^. In the case of few-layer h-BN, it was observed that the thermal conductivity decreased from 360 W/m-K to 250 W/m-K as the number of h-BN layers decreased from 11 to 5. This decreasing trend was also confirmed by micro-Raman spectroscopy experiments which reported a thermal conductivity of 243 W/m-K for suspended few-layer h-BN^34^. Nevertheless, our aim in the present work is to comparatively investigate the effect of grain size on the thermal conductivity of h-BN films, and not to predict the exact value of such conductivity. The results in Fig. 2 show a decreasing trend in the thermal conductivity with temperature, which is expected due to the increase in phonon-phonon scattering at higher temperatures^35^. Therefore, the thermal conductivity converges at lower correlation times as the temperature increases due to a reduction in phonon lifetimes. For pristine h-BN sheets at 900 K we found a thermal conductivity of 95 ± 10 W/m-K which is less than one third of that at room temperature.

**Figure 3:**
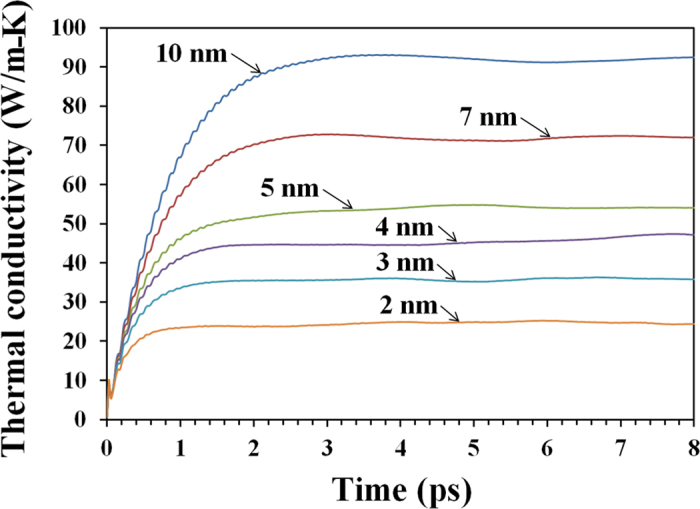
Calculated thermal conductivity of ultra-fine grained h-BN sheets as a function of correlation time at room temperature (300 K). Samples with smaller grains have more defects along the grain boundaries which decrease the conductivity.

Next, we analyse the thermal conductivity of polycrystalline h-BN films. In Fig. 3 we show the calculated thermal conductivity as a function of correlation time for h-BN membranes with average grain sizes from 2 nm to 10 nm at 300 K. We remind that in the construction of polycrystalline samples nucleation sites were distributed uniformly, originating samples with a uniform grain distribution. Furthermore, in order to have a representative volume element for each polycrystalline sample, we included a minimum of 16 grains in each sample. Therefore, the thermal conductivity of each sample is expected to be isotropic such that the calculated thermal conductivity is independent of the measuring direction. Indeed, simulation results confirmed that the thermal conductivity of our polycrystalline h-BN films is isotropic, and the conductivity values in Fig. 3 are the average over two in-plane directions and over several samples. We found that by decreasing the effective grain size the thermal conductivity also decreases, which is primarily due to the increase of defect concentration along the grain boundaries. Also, by decreasing the grain size the conductivity converges at lower correlation times, which indicates a reduction in phonon lifetimes.

**Figure 4:**
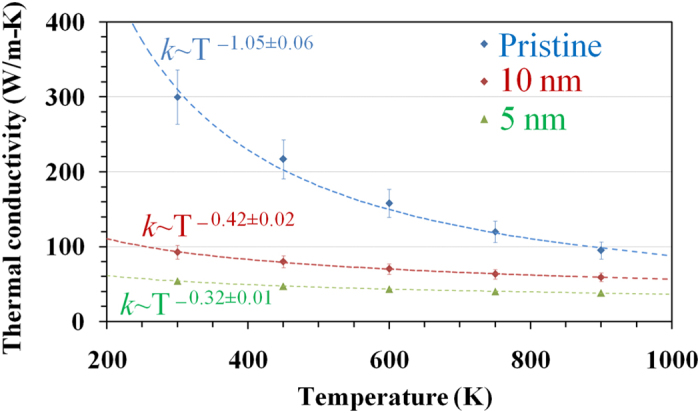
Temperature dependence of the thermal conductivity for monocrystalline and polycrystalline h-BN sheets. Deviation from inverse temperature dependence indicates an increase in phonon-defect scattering.

In order to quantify the contribution of each factor in the thermal conductivity of polycrystalline h-BN, we calculated the conductivity of samples with different grain sizes at a range of temperatures from 300 K up to 900 K. Figure 4 presents the temperature dependence of the thermal conductivity for monocrystalline h-BN, and polycrystalline films with grain sizes of 10 nm and 5 nm. For pristine h-BN films the thermal conductivity is inversely proportional to the temperature, which implies that the major contribution to the thermal resistance is due to phonon-phonon scattering. For samples with an effective grain size of 10 nm, the conductivity still decreases with temperature, but now it scales as ~*T*^−0.42^. This deviation from the inverse dependence observed in the pristine case is due to the contribution of phonon-defect scattering to the thermal resistance. When the grain size is reduced down to 5 nm, the number of defects in the sample increases, which then reduces the dependence of the conductivity to ~*T*^−0.32^. If the major contribution to the thermal resistance was due to phonon-defect scattering, there would be no temperature dependence in the thermal conductivity, since the number of defects does not increase with temperature (at least in the range considered here)^35^. Therefore, we can conclude that the reduction in thermal conductivity when the effective grain size is reduced, observed in Fig. 3 is indeed due to the increase in defects along the sample.

**Figure 5:**
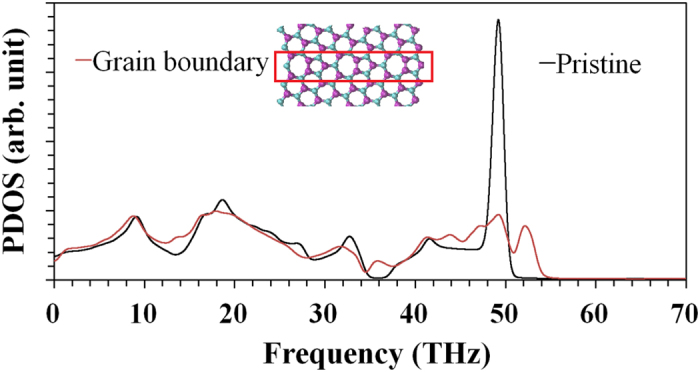
Vibrational density of states for pristine and polycrystalline h-BN sheets calculated at 300 K. The population of high-frequency modes (~49 THz) is reduced due to the defected structure along the grain boundary.

We also analysed the effect of the polycrystallinity on the vibrational spectrum of h-BN samples by comparing the phonon density of states (PDOS) of atoms in pristine sheet and also along a grain boundary. In Fig. 5 we observe a general decrease in the PDOS and a broadening of most peaks. In particular, there is a decrease in the PDOS for modes with frequency below 1 THz, which posses the longest wavelength and contribute significantly to the thermal conductivity of the material. The presence of grain boundaries and the associated defects also close the vibrational band gap present in the spectrum of pristine h-BN around 36 THz. Finally, the PDOS of high-frequency optical modes is considerably reduced in polycrystalline h-BN. Overall, the changes observed in the PDOS are consistent with a lower value for the thermal conductivity of polycrystalline h-BN, relative to pristine h-BN.

Molecular dynamics is a powerful tool to probe structural properties at the nanoscale. However, the high computational cost of atomistic MD simulations imposes limits on the investigation of materials at the macroscopic level, such as polycrystalline films with micrometer grain sizes. Therefore, in addition to atomistic modelling, we constructed polycrystalline models using the finite element (FE) approach, and then combined the results with the ones obtained from MD simulations.

**Figure 6:**
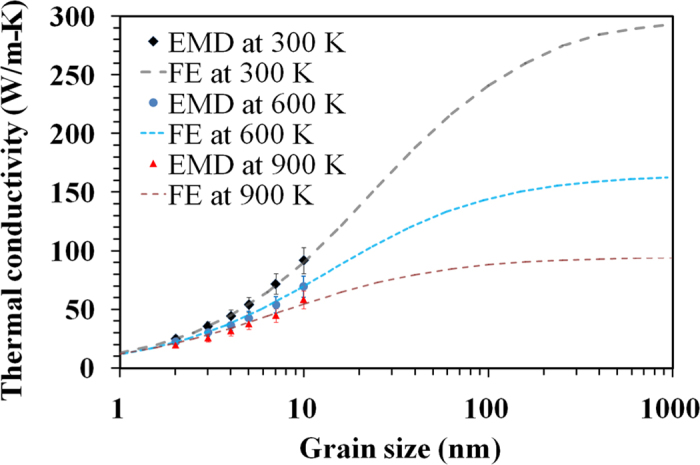
Thermal conductivity of polycrystalline h-BN sheets as a function of grain size at 300 K, 600 K and 900 K. Data points represent molecular dynamics results. Dashed lines are the finite element predictions for the thermal conductivity of polycrystalline h-BN films with larger grain sizes.

In Fig. 6 we plot the calculated thermal conductivity of polycrystalline h-BN as a function of grain size. The effective grain boundary thermal conductance in the FE models is obtained by fitting to the EMD data points. As shown, for ultra-fine grained samples the FE results match those obtained by EMD simulations. This observation confirms the validity of constructed continuum modelling. In this regard, our FE models predict an effective grain boundary thermal conductance of ≈11 ± 1 GW/m^2^K which is found to be independent of sample temperature. Our modelling shows that in spite of substantial reduction in the grain’s thermal conductivity at higher temperatures, the boundary thermal conductance remains stable since it is limited mainly by phonon-defect scattering. In addition, FE calculations for various temperatures reveal that polycrystalline h-BN films with an equivalent grain size of 500 nm could yield a thermal conductivity only ≈3% smaller than monocrystalline, defect-free sheets. This observation shows that the presence of grain boundaries in h-BN sheets has limited effects on the thermal conductivity of h-BN films, and that polycrystalline h-BN with an effective grain size larger than 500 nm retains a high thermal conductivity.

**Figure 7:**
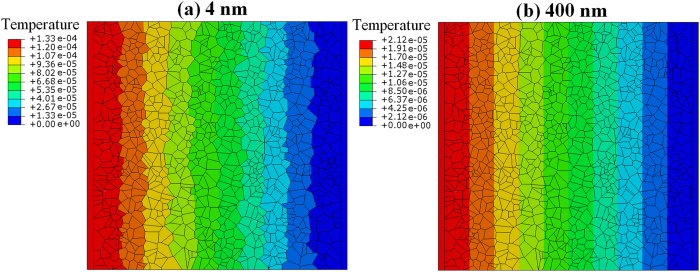
Comparison of established steady-state temperature profiles along polycrystalline h-BN sheets with equivalent grain size of 4 nm and 400 nm. The temperature is defined relative to the one assigned to the cold strip.

Figure 7 compares the temperature distribution in two samples with grain sizes of 4 nm and 400 nm, obtained by FE modelling. For the sample with an average grain size of 4 nm, the temperature inside the grains is relatively constant and temperature changes occurred mainly along the grain boundaries. This observation confirms that for ultra-fine grained structures the grain boundaries present the major contribution toward the thermal resistance of the sample. In this case, grains with larger length along the heat current direction carry the main portion of heat flux. On the other hand, for the sample with an equivalent grain size of 400 nm, the temperature changes occurred mostly inside the grains. In this case, the grain boundary contribution to the thermal resistance turns out to be much smaller and the heat is carried uniformly and independent of grain’s length.

**Figure 8:**
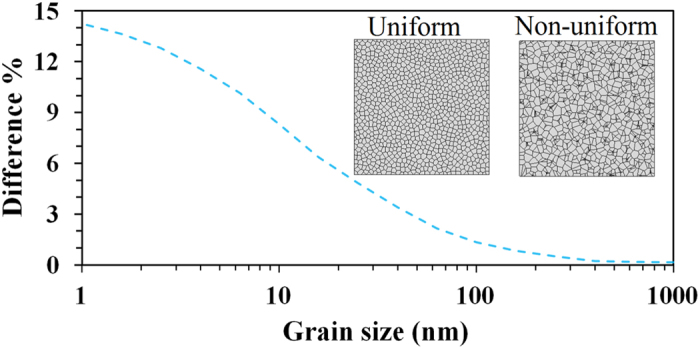
Difference in thermal conductivity of polycrystalline sheets with non-uniform grains relative to sample with uniform grain distribution at 300 K. The relative difference decreases with average grain size. Two finite element sample configurations are also shown.

In CVD grown polycrystalline sheets the grain distribution can be non-uniform^36^,^37^. In order to investigate the effect of non-uniform grains on the conductivity of polycrystalline sheets, we compared FE models with uniform and non-uniform grain configurations. Figure 8 presents the calculated relative difference in thermal conductivity of a non-uniform configuration with respect to a uniform configuration as a function of grain size at 300 K. The relative difference in thermal conductivity decreases as the average grain size increases. When the average grain size is very small (≈1 nm) our multiscale models predict a maximum relative difference smaller than 15%. Meanwhile, for an average grain size of 200 nm, the relative difference in conductivity is less than 1%. When the equivalent grain size reaches 500 nm, the relative difference is negligible. In order to account for this behaviour, we remember that in samples with ultra-fine grains the contact resistance between grains is the main factor determining the thermal conductivity of the sample, as shown in Fig. 7. In the case of a non-uniform grain distribution, heat is carried primarily through large connected grains forming a percolating path. Whereas if the grain distribution is uniform the heat flux is evenly distributed among grains. Therefore, samples with non-uniform grain distribution present higher effective thermal conductivity with respect to uniform samples. As the average grain size increases the number of grain boundaries decreases, and the influence of the boundary resistance in the thermal conductivity also decreases, such that the conductivity of polycrystalline sheets becomes less sensitive to the grain distribution. Furthermore, the role of boundary resistance was found to be more significant at 300 K such that at higher temperatures the effect of grain configuration would be less important.

**Figure 9:**
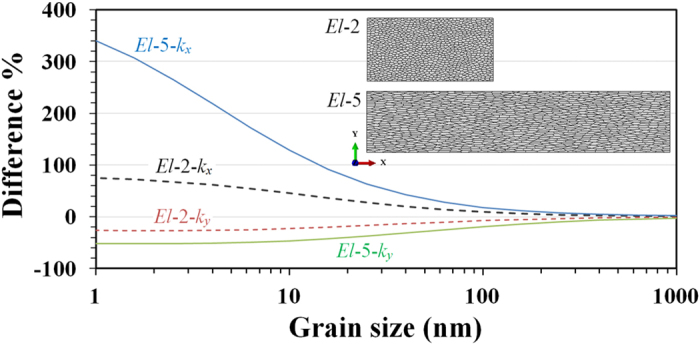
Difference in thermal conductivity along *x* and *y* directions for two elongation ratios (El-2 and El-5) relative to sample with uniform grain distribution at 300 K. The relative difference is larger along the elongated directions, but decreases with average grain size.

Finally, in CVD grown sheets, grain configuration can be modified by controlling the reactant flow rates, local environments and substrate properties during growth^38^,^39^,^40^. Therefore, we also investigated the thermal conductivity of polycrystalline h-BN films with elongated grains. These FE models were built by stretching a uniform structure in one direction (*x*-direction). The elongation ratio (stretching factor) is then equivalent to the grain’s aspect ratio (length to width ratio). We conducted simulations for two elongation ratios: 2 and 5. In Fig. 9 we show the relative difference in thermal conductivity of samples with elongated grains with respect to samples with uniform grains. The difference in conductivity was calculated along the elongated direction (*x*) and perpendicular to it (*y*). Our results reveal an increase in thermal conductivity along the elongated direction and a decreased along the perpendicular direction. The data also shows that the increase along the elongated direction is more pronounced than the decrease in the perpendicular direction, and that the effect of elongated grains on the thermal conductivity decreases with the grain size. These results are in agreement with our interpretation of the role played by the contact resistance between grains. In the case of samples with elongated grains, heat propagation along the longitudinal direction encounters fewer boundaries, and the sample presents a lower effective thermal resistance along this direction, which results in higher thermal conductivity along the elongated direction. Therefore, our results show that polycrystalline h-BN films with small grain sizes may present anisotropic thermal conductivity. Nonetheless, for an average grain size of 500 nm, the relative difference in thermal conductivity presented by samples with elongated grains is negligible.

## Discussion

The presence of defects in the crystalline structure of a material is expected to alter at least some of its intrinsic physical properties. In the case of large area graphene and h-BN samples grown by the CVD method, the presence of grains with different orientations is unavoidable. Grain boundaries present a range of topological defects which are expected to modify the physical properties of these materials. In terms of heat transport, defects reduce the thermal conductivity of the material, as shown for graphene recently^21^. Regarding the application of h-BN in heat management devices, due to its high thermal conductivity, it is important that the presence of defects do not hinder its ability to transfer heat efficiently. Our MD simulations show that as the effective grain size decreases, the thermal conductivity of h-BN becomes dominated by phonon-defect scattering rather than phonon-phonon scattering. Nonetheless, the combination of MD results with our FE modelling shows that polycrystalline h-BN films with an equivalent grain size of 500 nm present a thermal conductivity ≈3% smaller than monocrystalline, defect-free sheets from which we can conclude that he presence of grain boundary has limited effects on the thermal conductivity of h-BN films. Therefore, CVD grown h-BN sheets with an effective grain size larger than 500 nm are suitable for large area, high thermal conductivity applications, such as large thermal management devices.

In conclusion, using a combined atomistic-continuum modelling approach, we studied the heat conduction in polycrystalline h-BN films. We constructed large atomistic models of polycrystalline h-BN sheets with equivalent grain sizes ranging from 2 nm to 10 nm. We evaluated the thermal conductivity of defect-free and ultra-fine grained h-BN films at different temperatures using EMD simulations. To explore the thermal conductivity of samples with micrometer grain sizes, we constructed continuum models of polycrystalline sheets using the FE approach. Our modelling results confirm that for ultra-fine grained h-BN films the grain boundary resistance presents the major contribution to the thermal resistance of the sample. Nevertheless, by increasing the grain size the effect of boundary thermal resistance decreases substantially. Finally, our multiscale modelling shows that h-BN samples with grain sizes larger than 500 nm present thermal conductivity almost as high as pristine films, regardless of the grain distribution being uniform or non-uniform, being therefore suitable for high thermal conductivity applications.

## Methods

### Molecular Dynamics Simulations

We employed classical MD simulations to investigate the effect of grain size on the thermal conductivity of h-BN films. All MD simulations were performed using LAMMPS (Large-scale Atomic/Molecular Massively Parallel Simulator)^41^. The accuracy of the predictions from MD simulations depends on the use of appropriate potential functions to describe the atomic interactions. In this work, we employed the Tersoff potential^42^,^43^ with parameters obtained by Lindsay and Broido to model h-BN sheets^12^. The proposed potential could predict phonon dispersion curves of bulk h-BN in close agreement with experimental measurements^12^.

Equilibrium molecular dynamics was used to evaluate the thermal conductivity of h-BN films. Each structure was first relaxed to zero stress along the planar direction at room temperature using a Nosé-Hoover thermostat and a barostat for 25 ps^44^,^45^,^46^. The structures were then equilibrated at constant volume and room temperature using a Berendsen thermostat for another 25 ps^47^. Finally, before evaluating the thermal conductivity, we used removed all thermostats and let the system evolve at constant energy for another 25 ps.

The EMD method relates the ensemble average of the heat current auto-correlation function (HCACF) to the thermal conductivity *κ*, via the Green-Kubo expression:


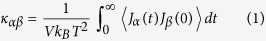


where *α*, *β* denote the three Cartesian coordinates, *k*_*B*_ is the Boltzmann constant, *V* and *T* are the volume and temperature of the system, respectively. In the calculation of system volume, we assumed a thickness of 0.33 nm for single layer h-BN membrane^48^. In the case of membrane-like materials such as graphene and h-BN, the conductivity is isotropic for in-plane directions. Therefore, we calculated the thermal conductivity as the average over two orthogonal in-plane components, i.e., *κ* = (*κ*_*xx*_ + *κ*_yy_)/2. The auto-correlation function of the heat current 〈*J*_*α*_(*t*)*J*_*β*_(0)〉 can be calculated using the heat current 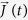 as expressed by^41^:


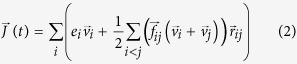


here, *e*_*i*_ and 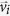 are the total energy and the velocity vector of atom *i*. In addition, 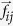 and 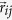 are the interatomic force and distance vector between atoms *i* and *j*, respectively. By performing constant energy simulations, the in-plane heat current was recorded to calculate the HCACFs. Several independent simulations were performed and the obtained HCACFs were averaged to calculate the effective thermal conductivity using Eq. (2).

The PDOS was calculated from the velocity autocorrelation function (VACF) by post-processing 100 ps trajectories, in which atomic velocities were printed out every 5 fs. The VACF was then normalised such that *VACF*(*t* = 0) = 1, and averaged over all atoms in a region. The final PDOS was obtained from the Fourier transform of the averaged VACF as


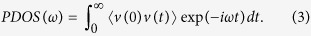


### Modelling Polycrystalline h-BN films

In order to construct atomistic models of polycrystalline h-BN films, we developed a program which assigned to every grain a nucleation site. These nucleation sites were uniformly distributed at random locations on a plane with predefined dimensions. In this work, we assumed an equivalent square geometry to define the grain size. Accordingly, the effective grain size for a particular structure is given by 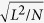, where *L* is the plane length and *N* is the number of grains (or nucleation sites) in that plane. Therefore, for a plane with 40 nm length and 16 grains the equivalent grain size would be 10 nm. For each nucleation site a random direction was assigned which defined the crystal orientation. Next, the growth of grains was simulated using an iterative process. In this approach, in each step a random grain was selected and the growth was applied. The growth of a grain from a boundary atom is terminated when it meets an atom from the neighbouring grain, which happens when the distance between the atoms is below 0.1 nm, or if it was already bonded with three atoms. The growth of the polycrystalline structure is finished when all grains meet their neighbours and there is no possibility for any additional atom to be included in the lattice. We note that the constructed models in this study were periodic in planar directions which is a requirement for the evaluation of thermal conductivity using the EMD method.

At this stage, the initial atomic positions are created, however the atomic configurations along the grain boundaries are not relaxed yet. In order to achieve relaxed grain boundaries, we used molecular dynamics simulations^19^,^20^,^21^,^22^. Because the Tersoff potential presents very limited reactivity, it cannot be used to relax the boundary structures. Therefore, we first replaced boron and nitrogen atoms with carbon atoms and then used the second-generation reactive empirical bond order (REBO) potential^49^ to model the atomic interactions during relaxation of the boundaries. The initial structure was equilibrated at room temperature (300 K) for 10 ps using a Nosé-Hoover thermostat. The structure was then heated up to 3000 K at constant volume for 30 ps. Next, the temperature of the structure was kept at 3000 K for another 30 ps. The large amplitude of atomic vibrations at 3000 K lets the atoms rearrange their positions to form relaxed grain boundaries and boundary junctions. Finally, the relaxed and equilibrated structure was obtained by cooling the system down to room temperature at constant volume for 10 ps. The final polycrystalline h-BN films were then acquired by replacing carbon atoms with the original boron and nitrogen atoms^22^.

### Finite Element Modelling

**Figure 10:**
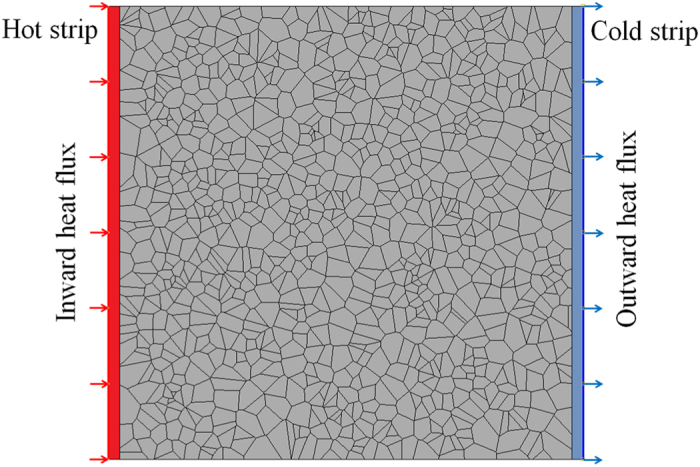
A typical constructed finite element model consisting of 1000 grains for the evaluation of thermal conductivity of polycrystalline h-BN films with large grain sizes. For the loading condition, we applied inward and outward surface heat fluxes on the outer surfaces of highly conductive strips.

In the FE models considered here, we assumed that grain boundaries present an effective thermal contact conductance. This effective thermal conductance was estimated by fitting the FE results to those of EMD for ultra-fine grained structures^21^. Similarly to our MD modelling, in the FE models we uniformly distributed 1000 points in a square plane with predefined dimensions. Based on the distributed points, we constructed Voronoi cells with mirror symmetry at all edges. The models were constructed in ABAQUS/Standard by python scripting. A sample of a constructed finite element model in ABAQUS consisting of 1000 grains is presented in Fig. 10.

For the evaluation of thermal conductivity, we included two highly conductive strips at the two ends of the structure, also shown in Fig. 10. In order to model the contact resistance of grain boundaries, we introduced contact elements between interacting grains. In this case, the master surface was chosen to be the one belonging to the grain closer to the hot strip and the other contacting surface (closer to the cold strip) was selected to be the slave surface. Regarding loading conditions, a constant inward heat flux (+*q*) was applied on the external surface of a strip (hot surface) while on the outer surface of the opposite strip (cold surface) the same magnitude outward heat flux (−*q*) was applied. In addition we also set the temperature of the outer surface of cold strip to zero as the initial value for our problem. As a result of applied loading condition, a steady-state temperature profile formed along the sample. The temperature difference along the structure, Δ*T*, was computed from temperature on the outer surface of the hot strip. The effective thermal conductivity, *κ*_*eff*_, was then calculated from the one-dimensional form of the Fourier law:


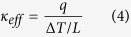


where *L* is the sample length.

## Additional Information

**How to cite this article**: Mortazavi, B. *et al.* Modelling heat conduction in polycrystalline hexagonal boron-nitride films. *Sci. Rep.* **5**, 13228; doi: 10.1038/srep13228 (2015).
